# Comparison of the Effects of External and Internal Lateral Nasal Osteotomies on Ecchymosis, Periorbital Edema, and Step off Deformity After Rhinoplasty

**DOI:** 10.29252/wjps.8.3.345

**Published:** 2019-09

**Authors:** Mahdi Gholami, Assila Vaezi

**Affiliations:** Department of Oral and Maxillofacial Surgery, Dental School, Mashhad University of Medical Sciences, Mashhad, Iran

**Keywords:** Rhinoplasty, Nasal osteotomy, Ecchymosis, Edema, Deformity

## Abstract

**BACKGROUND:**

Periorbital edema and ecchymosis are considered as the main complications of rhinoplasty. The current study was conducted to compare the effects of internal and external lateral osteotomies on ecchymosis, periorbital edema, and step off deformity after rhinoplasty.

**METHODS:**

This double-blind randomized clinical trial was conducted on 69 patients (including 37 and 32 cases undergoing external and internal lateral osteotomies, respectively), between the fall of 2016 and 2018. The participants were randomly divided into two groups and matched by gender and age for rhinoplasty. In addition, all surgeries were performed by the same surgeon to control the confounding variables. Edema, ecchymosis, and step off deformity were evaluated by a researcher blind to the methods of rhinoplasty 2, 7, and 21 days after the surgery.

**RESULTS:**

There was no significant difference between the two groups regarding postoperative edema on days 2, 7, and 21 post-operation. Similarly, the postoperative ecchymosis demonstrated no significant difference between the two groups on the 2^nd^, 7^th^, and 21^st^ days post-surgery. No significant difference was noted between the two groups in terms of step off deformity on day 21^st^.

**CONCLUSION:**

The obtained results demonstrated no significant difference between the internal and external lateral osteotomy techniques. Based on our findings, surgeons should apply a procedure with the lowest side effects according to their experiences.

## INTRODUCTION

Human likes to look beautiful; in this regard, inclination toward beauty is a long-standing desire in the nature of humans.^1^ Rhinoplasty is the most prevalent cosmetic surgery in the world, which is on a growing trend.^2^ This surgery is conducted owing to various motivations, the most common of which are the alteration of the facial appearance and correction of the respiratory tract.^3^ Nowadays, rhinoplasty is divided into two categories, including open surgery or external technique and close surgery or endonasal technique. In both techniques, postoperative edema and periorbital ecchymosis are unpleasant outcome for patients.^4^^-^^6^

Lateral osteotomy is one of the main parts of rhinoplasty in both open and close methods, which is performed due to the regeneration of the nasal form (pyramid shape).^6^ In the lateral osteotomy, the angular vessels and periosteum are usually damaged causing ecchymosis, as well as periorbital inflammation and edema.^7^ So it is considered as the predominant cause of postoperative edema and periorbital ecchymosis. However, this step is inevitable during rhinoplasty for narrowing the nasal pyramid in most cases.^8^^-^^10^ Lateral osteotomy can be performed through either percutaneous (external or perfusion-form methods) or linear endonasal method.^11^

 Several strategies have been applied in order to decrease the periorbital edema and ecchymosis These strategies include the use of steroids during and after the surgery, postoperative cold compress, point pressure, and plasters on the damaged area.^12^^-^^17^ It has been declared that a single-dose of dexamethasone reduces postoperative edema and ecchymosis.^15^ However, the injection of lidocaine and epinephrine (1:100,000) before lateral osteotomy reportedly resulted in no significant effects.^15^ Moreover, it has been proven that several high-dose injections of corticosteroids reduced the postoperative edema and ecchymosis.^16^ Based on the evidence, the application of cold compress around the periorbital region and nasal area, mitigated the periorbital edema and ecchymosis.^17^ In spite of the proposed solutions, none of them can definitely restrict the associated complications and edema and periorbital ecchymosis are still the main concerns.^12^^-^^20^

It has been indicated in several studies that endonasal lateral nasal osteotomy resulted in lower extents of edema and periorbital ecchymosis than the external method.^18^^-^^20^ However, based on a number of investigations, the external nasal osteotomy is superior to internal osteotomy.^21^^-^^24^ Given the controversial results regarding this issue, the present study was conducted to evaluate the effects of internal and external lateral nasal osteotomies on periorbital edema, ecchymosis, and step off deformity. 

## MATERIALS AND METHODS

This double-blind randomized clinical trial was conducted on 69 patients (including 37 and 32 cases subjected to external and internal lateral nasal osteotomies, respectively) undergoing rhinoplasty between the fall of 2016 and fall of 2018. The study was approved by the Ethics Committee of Mashhad University of Medical Sciences, Mashhad, Iran (IR.MUMS.SD.REC.1394.239). Patients without history of nasal trauma, systemic disorders and anticoagulant consumption who were candidates for primary rhinoplasty were enrolled in the study. All subjects signed the informed consent. The patients who were reluctant to follow up visits were excluded from the study. 

The patients were randomly divided into two groups and matched based on gender. For the purpose of group allocation, papers labeled A or B were placed inside a box. Label A represented external osteotomy, while label B denoted internal osteotomy. The patients selected the labels randomly; subsequently, they were visited by the surgeon based on the random selection method. Similar surgical conditions, anesthesia, and medications were considered for all patients. Furthermore, rhinoplasty surgeries were performed by the same surgeon for all patients in order to control the confounding factors. 

Surgical operation was conducted under general anesthesia induced with halothane, fentanyl, and nesdonal; however, corticosteroid was not totally injected. In the lateral osteotomy, adrenalin (1/100,000), along with lidocaine (1%), was injected in the medial and lateral side of the frontal process of the maxilla, 10 min before lateral osteotomy. Edema, ecchymosis, and step off deformity were evaluated by a researcher who was blind to the method of osteotomy, according to three scoring systems in the 2^nd^, 7^th^ and 21^st^ days after the surgery. The scoring system applied for edema evaluation was as follows:^25^ Grade 1: No coverage of the iris with the eyelids, Grade 2: Slight coverage of the iris with the swollen eyelids, Grade 3: Full coverage of the iris with the swollen eyelids, and Grade 4: Full closure of the eyes.

The scoring system used for evaluation of ecchymosis included Grade 1: No ecchymosis on the lower and/or upper eyelid, Grade 2: Ecchymosis up to the medial one-third of the lower and/or upper eyelid, Grade 3: Ecchymosis up to the medial two-third of the lower and/or upper eyelid and Grade 4: Ecchymosis up to the full length of the lower and/or upper eyelid. In addition, the scoring system adopted for evaluation of step off deformity was as follows: Grade 1: No visible or touchable step at the nasal side wall, Grade 2: Touchable step at the nasal side wall, and Grade 3: Visible and touchable step at the nasal side wall. The data were analyzed using SPSS software (version 20, Chicago, IL, USA). The independent t-test was used to compare the normally distributed quantitative variables; otherwise, Mann-Whitney U test was applied. In addition, the evaluation of the two qualitative variables was accomplished using the Chi-Square test. P value less than 0.05 was considered statistically significant.

## RESULTS

The present study involved the investigation of 69 subjects (including 37 and 32 patients undergoing external and internal lateral nasal osteotomies, respectively). The mean ages of the patients in the external and internal lateral osteotomy groups were 29.08±6.51 and 27.81±6.13 years, respectively. The results showed no statistically significant difference between the two groups in terms of age (*p*=0.4). The external osteotomy group included 13 (35.1%) males and 24 (64.9%) females and the internal osteotomy group consisted of 8 (25%) males and 24 (75%) females. The obtained data demonstrated no significant difference between the two groups regarding gender distribution (*p*=0.2).

 In the external osteotomy group, the mean scores of edema were 1.51±0.73, 1.14±0.34, and 1.05±0.22 on the 2^nd^, 7^th^, and 21^st^ day post-operation, respectively. Regarding the internal osteotomy group, these scores were obtained at 1.41±0.49, 1.06±0.24, and 1.06±0.24 on the aforementioned days, respectively. There was no significant difference between the two groups regarding post-operative edema on days 2 (*p*=0.8), 7 (*p*=0.3), and 21 (*p*=0.8). The mean edema scores reduced from day 2 to days 7 and 21 in both groups (*p*=0.01 for both groups); however, the post-hoc test demonstrated no significant difference in pair comparisons (Table 1).

**Table 1:** Mean score of edema 2, 7, and 21 days after rhinoplasty in external and internal lateral osteotomy groups

**Score of edema**		**Number**	**Mean**	**SD**	**Min**	**Max**	***p*** value*
2 days after rhinoplasty	External lateral osteotomy	37	1.51	0.73	1	3	0.8
	Internal lateral osteotomy	32	1.41	0.49	1	2	
7 days after rhinoplasty	External lateral osteotomy	37	1.14	0.34	1	2	0.3
	Internal lateral osteotomy	32	1.06	0.24	1	2	
21 days after rhinoplasty	External lateral osteotomy	37	1.05	0.22	1	2	0.8
	Internal lateral osteotomy	32	1.06	0.24	1	2	

*Man-Whitney U statistical test

The mean scores of postoperative ecchymosis were 3.24±1.06, 2.97±1.11, and 1.30±0.52 on the 2^nd^, 7^th^, and 21^st^ days post-operation in the external osteotomy, respectively. These values were respectively estimated at 3.38±1.07, 2.84±1.24, and 1.41±0.71 in the internal osteotomy group on the aforementioned stages. The statistical analysis of postoperative ecchymosis demonstrated no significant difference between the two groups in this regard; 2 (*p*=0.4), 7 (*p*=0.7), and 21 (*p*=0.7) days after surgery. The mean scores of ecchymosis significantly decreased in both groups (*p*=0.01) from day 2 to days 7, and 21. Moreover, the obtained data showed that the ecchymosis score significantly reduced on the 21^st^ day in comparison to those obtained on the 2^nd^ and 7^th^ days after surgery (Table 2). 

**Table 2:** Mean score of ecchymosis 2, 7, and 21 days after rhinoplasty in external and internal lateral osteotomy groups

**Score of ecchymosis **		**Number**	**Mean**	**SD**	**Min**	**Max**	***p*** value*
2 days after rhinoplasty	External lateral osteotomy	37	3.24	1.06	1	4	0.4
	Internal lateral osteotomy	32	3.38	1.07	1	4	
7 days after rhinoplasty	External lateral osteotomy	37	2.97	1.11	1	4	0.7
	Internal lateral osteotomy	32	2.84	1.24	1	4	
21 days after rhinoplasty	External lateral osteotomy	37	1.30	0.52	1	3	0.7
	Internal lateral osteotomy	32	1.41	0.71	1	3	

*Man-Whitney U statistical test

The mean step off deformity score was not evaluated on the 2^nd^ day after surgery due to presence of the nasal splint. However, the mean values of this variable on days 7 and 21 post-surgery were 1.57±0.50 and 1.24±0.43 in the external osteotomy group, respectively. Regarding the internal osteotomy group, the mean scores were obtained at 1.28±0.45 and 1.09±0.29 on days 7 and 21, respectively. Statistical analysis demonstrated no significant difference between the two groups in terms of step off deformity on day 21 (*p*=0.1). Nonetheless, the internal osteotomy group showed a significant reduction in the mean score of this variable in comparison to the external lateral osteotomy group on day 7 (*p*=0.01). Additionally, the mean step off deformity score significantly declined in both groups on day 21 (*p*=0.001), compared to that obtained on day 7 post operation (Table 3).

**Table 3:** Mean score of step off deformity 2, 7, and 21 days after rhinoplasty in external and internal lateral osteotomy groups

**Score of steps**		**Number**	**Mean**	**SD**	**Min**	**Max**	***p*** value*
7 days after rhinoplasty	External lateral osteotomy	37	1.57	0.50	1	2	0.01
	Internal lateral osteotomy	32	1.28	0.45	1	2	
21 days after rhinoplasty	External lateral osteotomy	37	1.24	0.43	1	2	0.1
	Internal lateral osteotomy	32	1.09	0.29	1	2	

*Man-Whitney U statistical test

## DISCUSSION

Based on the obtained data, there was no statistically significant difference between the two groups in terms of the post-operative step off deformity score. However, the patients in the internal lateral osteotomy group showed a significant reduction in the mean score of this variable on day 7, in comparison to the external lateral osteotomy group. Despite the wide scope of the studies investigating the post-operative edema and ecchymosis, there are limited investigations comparing the step off deformity between the internal and external lateral osteotomy methods. 

Hashemi *et al*.^26^ examined the step and medialization on each side after removal of the split and charted, if there was any difference between the two sides; nonetheless, they observed no significant difference between the internal and external methods in this regard. Our results revealed that the mean step off deformity score was not significantly different between the two groups, 21 days after the operation. Further investigations demonstrated that the mean edema and ecchymosis scores were not significantly different between the two groups on days 2, 7, and 21 after rhinoplasty. 

Motamed *et al*.^27^ compared internal and external osteotomies in terms of edema and ecchymosis. They assessed 168 patients 1, 3, 7, and 30 days after rhinoplasty and observed no significant difference between the two methods regarding these two variables, which is in line with our investigation. In a study conducted by Van loon,^28^ edema was assessed in both internal and external methods by three-dimensional stereophotogrammetry. However, the obtained data showed no significant difference between the two groups in this regard, which is consistent with our findings. In another study performed by Yücel^29^ on 20 patients, the scores of edema on days 2 and 7 post-operation in both external and internal groups were equal. 

Edema and ecchymosis affect the results of rhinoplasty and cause fear and dissatisfaction among patients, relatives, and surgeons. Various methods have been suggested for lateral osteotomy to reduce the post-operative complications. However, there is no agreement on the superior method, and the surgeons have different opinions in this regard. Some researchers, such as Rees *et al*.^21^ and Ford *et al*.,^23^ are in favors of the external technique. They believe that the external method reduces trauma in the soft tissue, nasal mucosa, and periosteum.^21^^-^^23^

Yazdani *et al*.^30^ evaluated the rupture in the nasal mucosa, edema, and ecchymosis in the internal and external osteotomy techniques. The results showed that the complications and severity of rupture in the nasal mucosa, edema, and ecchymosis decreased in the external group; however, this reduction was not statistically significant. In a study carried out by Giacomarra *et al.*, the external method was preferred over the internal one due to inducing less mucosal damage.^31^

Yücel investigating 20 patients showed that ecchymosis score significantly increased in the internal group; however, no statistically significant difference was observed in this regard, 7 days post-operation.^29^ In a study conducted by Hashemi *et al*.^26^ on 30 patients, edema and ecchymosis scores reduced one day after the external method. In the mentioned study, the severity of ecchymosis significantly reduced 7 days after surgery in the patients subjected to the external method. Nevertheless, ecchymosis severity significantly increased in the female group in comparison to that in the male group. 

On the other hand, Denneny and Tardy investigating the internal method using a 2-3 mm osteotome without a protector reported that the internal method reduced the edema, ecchymosis, and mucosal damage.^32^ There are many contradictory findings in this regard; accordingly, no consensus were preferred for the lateral nasal osteotomy method by surgeons. Generally, the researchers advocating the external methods believe that this method protects the periosteum, facilitates better control over the bone fractures, and obstructs nasal collapse and intranasal problems. Moreover, due to inducing lower tissue trauma, this method caused lower rates of bleeding, ecchymosis, and edema.^22^ On the other hand, the internal method advocators believe that this method, when implemented with a high precision, causes lower extents of edema and ecchymosis.^33^

However, there are effective factors in the incidence of post-rhinoplasty complications that should be investigated. These factors include the administration of nonsteroidal anti-inflammatory drugs, oral contraceptive pills, coagulation disorder, and blood pressure. In the current study, the role of confounding factors was reduced as much as possible, and both methods were evaluated under the same conditions. In this regard, similar surgeon, anesthetic method, equipment, drugs, and perioperative and postoperative situations were considered for all patients. 

Another point to be considered is the surgeon’s skill or experience in rhinoplasty that can affect the results. In this respect, the selection of the lateral nasal osteotomy method mostly depends on the surgeon’s experience than on his/her scientific information. The obtained results demonstrated no significant difference between the two methods; therefore, the surgeons are recommended to select a method that is better, easier, and less complicated according to their experience and discretion. In addition, further studies are recommended to confirm the findings of this study with a higher sample size using multi-centered investigations. It is also required to perform further investigations about the effective factors in rhinoplasty in order to plan for a new therapeutic strategy.
